# Analysing postprandial amino acid responses in crossover studies with the aaresponse package for R

**DOI:** 10.1007/s00726-024-03386-6

**Published:** 2024-03-21

**Authors:** Ron Wehrens, Jasper Engel, Jurriaan Mes, Aard de Jong, Diederik Esser

**Affiliations:** 1 Biometris, Wageningen University and Research, Droevendaalsesteeg 1, 6708 PB Wageningen, The Netherlands; 2 Wageningen Food and Biobased Research, Wageningen University and Research, Bornse Weilanden 9, 6708 WG Wageningen, The Netherlands

**Keywords:** Protein digestibility, Amino acid levels, Blood samples, Data analysis, Statistical software

## Abstract

Nowadays, a healthier and more sustainable lifestyle is the subject of much research. One example is the use of crossover trials to investigate the uptake of proteins, usually from alternatives to animal-based sources, by healthy volunteers. The data analysis is complex and requires many decisions on the part of the scientists involved. Such a process can be streamlined and made more objective and reproducible through bespoke software. This paper describes such a software package, $\textbf{aaresponse}$, for the $\textsf{R}$ environment, available as open source. It features ample visualization functions, supports consistent curation strategies, and compares amino acid uptake of different protein meals (interventions) through the use of mixed models analysing parameters of interest like the area under the curve (AUC). The defining feature is the use of parametric curves to fit the amino acid levels over time, increasing the robustness of the approach and allowing for more strict quality control strategies.

## Introduction

In the search for a more sustainable way of life, much attention is focused on plants as an alternative source of proteins. Not all plant proteins can be taken up easily by the human body, and much research has been devoted to assess digestibility characteristics of plant proteins into amino acids (AAs). The nine essential or indispensable amino acids (EAAs: phenylalanine, valine, threonine, tryptophan, methionine, leucine, isoleucine, lysine, and histidine) are of special importance, since humans cannot synthesize these themselves.

Several methodologies have been developed to report protein digestibility. The Protein Digestibility-Corrected Amino Acid Score (PDCAAS) assesses food protein quality taking into account human requirements (by focusing on the limiting type of amino acid) and digestibility, determined over the total digestive tract. In general, PDCAAS estimates are too optimistic, and better estimates are obtained by the more recent Digestible Indispensable Amino Acid Score (DIAAS) (FAO Expert Consultation 2013), adopted by the FAO/WHO and by the US FDA as the preferred best method for determining protein quality. Both methods, however, require animal experiments, and form less-than-perfect models for human digestion. Whereas in vitro models may partly replace animal studies (Butts et al. 2012), the results are still not good enough for a reliable comparison of the food values of different protein sources. Finally, true amino acid digestibility in humans can be measured using stable-isotope techniques, but this is rather challenging from a methodological perspective and, in addition, expensive.

A much simpler and yet direct approach is to measure postprandial amino acid concentrations in blood to estimate the digestibility potential of a protein source, and this is the technique we are focusing on in the current paper. Note that the levels of amino acids in blood only partially reflect the digestibility of a protein source: they are influenced by many factors, including breakdown and endogenous synthesis. The latter factor is not present for the EAAs, which therefore constitute a valuable measure for protein quality comparisons.

To compare protein digestibilities with a reference protein, typically crossover experiments are performed, in which healthy volunteers are given different protein meals, separated by a significant time so that carryover effects are avoided (see, e.g. Mes et al. 2022; Wegrzyn et al. 2022; Liu et al. 2019). During several hours after the meal, blood samples are taken and analysed for amino acid levels. Statistical analysis of these time course data then allows to draw conclusions about amino acid kinetics and protein uptake, indicated by parameters of interest (PoIs), typically AUC, the area under the curve, peak height, and the speed of uptake (the time to the peak maximum). Whereas these values per se may not be easy to interpret, comparison with a reference protein, often easily digestible, allows one to put things in perspective and, e.g., set up a ranking of the digestibility of proteins from different sources. When the same reference is used in different studies, the corresponding results can be directly compared, even when the individual studies differ in their design or measurement setup.

Data analysis for such experiments can be done in many ways, but is certainly not trivial. This paper proposes a simple and principled approach, implemented in open-source software, and comprising several steps. The first, to be executed for all amino acids in all participants separately, is to obtain estimates for the PoIs. Alternatively, one can also focus on AA totals, either considering all AAs, only the EAAs, or a specific AA subset. The simplest possible approach would approximate peak height by the differences between the highest and lowest values in each time series and take the time of the largest value as the time to the peak maximum. A parameter like AUC can be assessed by the trapezoid rule. These estimates, however, are very sensitive to noise and artefacts in the data. A more sophisticated strategy, less affected by experimental variation, is to fit parametric curves through the amino acid time series, and to extract estimates of the PoIs from the estimated curve parameters.

In the second analysis step, these PoIs are compared between the different proteins in the experiment. This can be done in several different ways, the simplest being an analysis of variance. A more powerful approach is based on linear mixed models. Their benefits include being applicable in unbalanced situations, and being much more flexible in the description of the model and the contrasts of interest.

In such an analysis, quality checks are needed at several stages to prevent individual outlying observations to have a large effect on the eventual outcome of the experiment. Sometimes, the raw data show individual measurements that deviate quite dramatically, or points showing a behaviour that is consistent across amino acids, but inconsistent in time, indicating that perhaps a sample is compromised. In other cases, strange values are observed at the level of the PoIs (e.g. implausible peak heights, maxima at the very first, or very last time point). The curve fits used to describe the amino acid time courses provide an intermediate possibility for curation: it is usually feasible to define ranges of suitable values for curve parameters, and to automatically flag cases that are outside these ranges for further inspection.

This paper describes a principled approach to analysing the data of protein digestibility experiments. To make this widely accessible, we have provided an implementation as an open-source package for the $\textsf{R}$ language (R Core Team 2022), $\textbf{aaresponse}$. The package includes several visualization tools for quality assessment and curation of the data as well as intermediate results, curve fit procedures to describe the amino acid time courses, and mixed-model functionality to compare proteins. Although developed with protein digestibility in mind, the package can also be used to analyse data from other types of nutrient-uptake experiments (glucose, fat, micronutrients,...) for which time course data are available.

## The aaresponse package for R

**Fig. 1:**
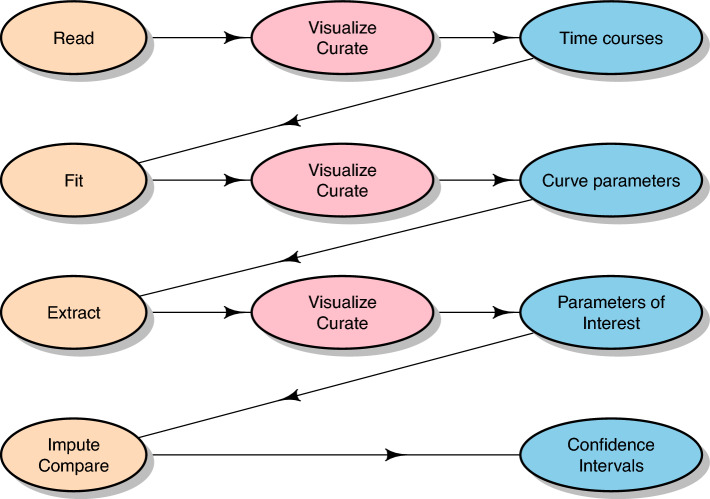
Flowchart of the analysis strategy in $\textbf{aaresponse}$. The left column contains the main steps, the middle column contains curation and associated visualization steps, and the right column contains the results of each step

This section describes the $\textbf{aaresponse}$ package, written in $\textsf{R}$, and developed for the analysis of protein uptake experiments using crossover designs. The methodology has been developed and used in several projects leading to peer-reviewed papers (Mes et al. 2022; Esser et al. 2023; van Dam et al. 2023; Ummels et al. 2023; Roelofs et al. 2024). The examples focus on a data set from one of these, which is distributed with the package and analysed more rigourously elsewhere (Esser et al. 2023). The $\textsf{R}$ code leading to the plots and tables is discussed in the $\textsf{R}$ package “vignette”, basically a walkthrough to obtain the results. An overview of the analysis strategy (and the package) is given in Fig. 1—note that at any point in time, earlier results may be revised by the user, and the analysis sequence can be continued from that point onwards (such feedback loops have not been included in the figure to avoid clutter). The following paragraphs describe the main functionalities of the package in more detail.

### Data visualization

Data coming from crossover experiments are complex: typically 10–20 participants are given protein meals in two to three periods, and eight to ten blood samples are taken and analysed for up to 20 amino acids or amino acid aggregates. This leads to thousands of data points. An appropriate visualization of the raw time courses is very powerful, in that it very quickly gives an idea of data quality, differences between proteins, differences between participants, and differences between amino acids. It also can point to anomalies, such as outlying measurements, and unexpected patterns: in one case, we were able to immediately spot a potential label mix-up which was later confirmed by going back to the original laboratory data. The $\textbf{aaresponse}$ package contains several standard visualizations for amino acid time courses, allowing users to easily zoom in on areas of interest. In addition, several types of principal component analysis (PCA, Jackson 1991; Wehrens 2020), allowing a multivariate view of the data, are supported.

### Fitting curves for amino acid time courses

Manual analysis of the amino acid time courses is labour-intensive, prone to errors, and, perhaps most importantly, not very robust against outlying observations. In our experience, these occur quite frequently, so our data analysis pipeline should be able to handle them. It is easy to imagine other potential problems—even the level of the baseline is hard to estimate in many cases. The crucial advantage of fitting a curve with a predefined shape through such data is that it includes an inherent smoothing, basically using information from all time points to determine appropriate curve parameters. The approach using curve fitting has a couple of additional advantages:

1. time points need not be spaced equidistantly, and may even vary within the experiment;
2. individual deviating values usually only have a minor effect on the parameters of a fitted curve;
3. missing values are allowed;
4. curves can be fit even when the values have not completely returned to baseline;
5. PoIs can be calculated analytically from the curve parameters.

**Fig. 2:**
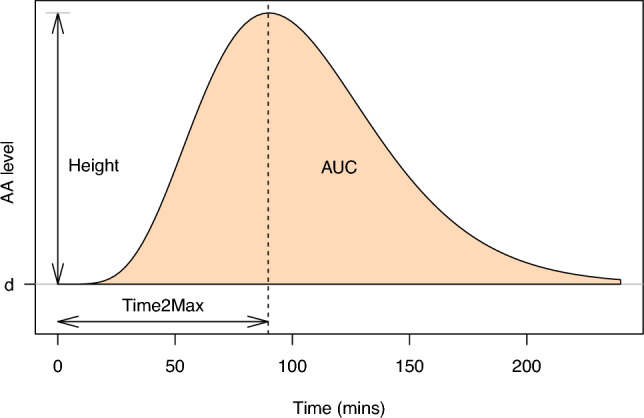
A Wood curve and the parameters extracted from it. In this case, the AUC value is calculated up to 4 h

Of course, the parametric form of the curve needs to be chosen carefully. A suitable model is given by the Wood curve, originally used to describe lactation behaviour in cattle (Wood 1967):

$$\begin{aligned}y(t) = d + at^{mc} e^{-ct},\end{aligned}$$

where *y*(*t*) is the level of a particular amino acid at time *t*. An example is shown in Fig. 2. Parameter *d* is the baseline, not present in the original paper by Wood, but added in other publications (see, e.g.  Engel 2003). Parameter *a* is related to the height of the peak, and the other parameters determine the shape of the curve. Parameter *m*itself determines the time to the peak maximum, and *mc* (in Wood’s publications indicated as *b*) is related to the increasing part of the curve; *c* itself describes the return to the starting position. Since the curve parameters in themselves have meaning, it can be useful to explicitly inspect them, e.g. to identify cases where curve fitting has led to unexpected results. Their values may even be a topic of study in particular situations.

Note that many other methods can be used for drawing smooth curves through a series of points (e.g., splines): the Wood curves employed here have the advantage of providing interpretable curve parameters, as well as having analytical solutions for the PoIs. Moreover, they lead to curves that always return to the fitted baseline (although this may happen only after the last measurement), something that with more flexible curve fitting methods like splines is not necessarily the case.

If there would be no baseline ($d=0$), the model could be fitted by taking logarithms and applying simple least-square regression. Here, we have to take account of the fact that all participants will have a non-zero (and different) baseline, corresponding to different AA levels in the sober state in which they start the experiment. This necessitates an optimization approach for finding the optimal values of the model parameters. In $\textbf{aaresponse}$, a nonlinear least-squares approach is used, performing 500 optimizations with different random starts and choosing the eventual best result.

Given that four parameters are estimated (*a*, *m*, *c* and *d*), we need to have at least five time points to obtain a fit in the first place, and preferably eight or more for a reliable result. However, even with a reasonable number of time points, it is important to assess the qualities of the fits. In some cases, the data may not conform to the Wood curve: in practice, one may see, e.g., amino acid levels that drop below the starting value, either due to natural variability or through metabolic processes not directly related to the experiment. In such a case, the fitted curves will be a compromise, usually putting the estimated baseline somewhere between the starting and ending values. Whether this is a useful approximation needs to be assessed on an ad hoc basis. Other situations potentially causing problems in curve fitting are very narrow peaks, e.g. containing just one point above the baseline, or completely flat profiles.

The optimization process will limit the ranges of possible values for the curve parameters, but it is unwise to choose them very narrow—that could lead to a failure to converge. It is better to start with liberal ranges, and to narrow down to plausible values in a subsequent curation step. Any values outside these plausible ranges can be replaced by $\texttt {NA}$, the $\textsf{R}$ coding for “not available”. If needed, one can always repeat the fit for specific cases with more narrow boundaries. If these plausible ranges are not known, or for some reason not appropriate, one can always revert to visual inspection: by plotting the sorted values for each of the four curve parameters one can easily identify any extreme observations, and these in turn can be related to specific time courses, which again can be inspected. Here, we only report results obtained with automatic curation using the defaults of $\textbf{aaresponse}$, eliminating nothing but the most glaring outliers—by necessity, defined by the user. However, it is important to note that it is actually preferable to err on the safe side: removing one time curve too many will likely not influence the result very much, whereas including a really strange observation may wreak havoc.

### PoIs

Once we have the curve estimates, the PoIs follow analytically (Rook et al. 1993). The three PoIs we consider here are AUC, peak height, and time to peak maximum (see Fig. 2). We have already seen that the time to the peak maximum ($\texttt {Time2Max}$) is given by *m*; the AUC value, relative to the baseline *d*,^1^ is given by

$$\begin{aligned} {\texttt {AUC}} = {a}/{c^{(b+1)}\Gamma (ct_{f},b+1)}, \end{aligned}$$

where $\Gamma (x,q) = \int _{0}^{x} z^{q-1}e^{-z} \,dz$ is the lower incomplete gamma function and $t_f$ the time point up to which the AUC value should be computed. The peak height of curve *y*(*t*) (relative to baseline *d*) is given by

$$\begin{aligned} {\texttt {Height}} = \max (y(t)) - d = a(b/c)^{b}e^{-b}. \end{aligned}$$

Also here, the PoIs may be compared to plausible ranges, or potential outlying values may be identified by visual inspection. Note that plausible ranges may depend on the type of variable—for amino acid totals, e.g., one would expect much larger peak heights and AUC values than for individual amino acids. Again, in cases where the values are clearly not within plausible regions, they could be replaced by $\texttt {NA}$.

### Imputation

In some cases, no suitable fit can be found, either because the curve fitting optimization does not converge, or because the curve parameters or PoIs are outside the optimization boundaries. In such cases, values will be stored as missing ($\texttt {NA}$) and will be ignored in the subsequent statistical analysis. It can be argued, however, that a major cause of these missing values is no uptake by the participant, or an uptake that is too low to measure, and that these missing values are actually highly informative. If that is the case, very low values for AUC and peak height would be more appropriate than missing values.

The default analysis considers only cases without missing values (responders), and this should be clearly stated to avoid incorrect interpretation of the results. An alternative is to impute the missing values. For the time to the peak maximum, this is hard to imagine (if there is no peak, where is the maximum?), but for AUC and peak height, estimates based on the distribution of the raw data are shown to be highly effective, especially for low-amplitude time courses. Basically, the idea is to use the 0.2 and 0.8 quantiles of the raw data (basically a robust estimate of the spread in the time course for one amino acid in one participant) to set up a regression model for either peak height or AUC value. After the imputation, the comparison of protein interventions can proceed in the normal way, now presenting results for the whole population (responders as well as non-responders). Note that with large numbers of non-response, the assumptions of the mixed model may be violated, so this needs to be checked. In any case, it is advisable to perform analyses both including and excluding non-responders, and to compare the results: where the differences are large or unexpected, further inspection is needed. For more details, we refer to the vignette included in the $\textsf{R}$ package.

### Further statistical analysis

In some cases, simply obtaining good estimates of the PoIs and the corresponding confidence intervals is the end goal of the experiment. However, often one is interested in comparisons, e.g. between different protein meals. The statistical analysis in the final step of the $\textbf{aaresponse}$ pipeline is geared towards digestibility of different proteins, both in terms of completeness and speed of uptake. Note that many more analyses are possible, and are usually easily implemented in the $\textsf{R}$ environment. Here, we focus on the comparison of one or more proteins with a reference protein.

For every PoI, the same approach is followed. The first basic step is to average the PoIs over all participants, leading to tables with averages and standard deviations. An analysis of variance could be used to investigate whether there are differences between different protein sources, but as stated in the introduction section, linear mixed models are more suited here. These are fitted describing the PoI, in the simplest possible form, as a function of the protein meal and the participant:

$$\begin{aligned} {\texttt {PoI}} = {\texttt {Intervention}} + (1 \mid {\texttt {Participant}}). \end{aligned}$$

In this case, we are not interested in separate coefficients for participants, but rather in the variability in the responses of the participants. This is formalized by taking $\texttt {Participant}$ as a random effect—we use the Wilkinson–Rogers notation employed by the $\textbf{lme4}$ package (Bates et al. 2015), hence the $(1 \mid {\texttt {Participant}})$ term in the model definition. Parameters such as $\texttt {Intervention}$ (describing the protein meal) are fixed effects: here we *are* interested in the coefficients.

The equation can be extended in several different ways. Often, it is wise to include a fixed factor for $\texttt {Period}$, to catch any time dependencies—this is also the default in $\textbf{aaresponse}$. We do not expect such effects to be significant, since the different protein meals are consumed far apart in time, and carryover or learning effects are very unlikely, but it is good to check. In the formulation mentioned above, standard model assumptions hold: residuals are independently and identically distributed according to a normal distribution, and independent from the participant effects. More complicated mixed models allowing, e.g., different varibilities for different proteins, can be fit as well. The resulting model contains coefficients and confidence intervals for all protein PoIs. In addition, specific contrasts may be defined, by default comparing a test protein with the reference protein. This has been implemented using an *F*-test with the Kenward–Roger approximation (Kenward and Roger 1997).

The final decision concerns the type of contrast that is employed: for peak height, and the time to peak maximum, this is commonly the difference. For AUC, however, often the ratio is investigated. In $\textbf{aaresponse}$, both differences and ratios are supported—the latter is the default for AUC, and the former for the other two PoIs. In the plots, the line of “no difference” will be set accordingly: i.e., at zero for differences, and at one for ratios.

### Implementation and availability

The $\textbf{aaresponse}$ is written in $\textsf{R}$, using several packages for specific elements of the workflow. The main ones are the following: fitting Wood curves is done using package $\mathbf {nls.multstart}$ (Padfield and Matheson 2020), the mixed models for comparing different protein interventions are due to $\textbf{lme4}$ (Bates et al. 2015), supported by the $\textbf{emmeans}$ (Lenth 2023) and $\textbf{multcomp}$ (Hothorn et al. 2008) packages, and the graphics are based on $\textbf{lattice}$ (Sarkar 2008). The $\textbf{aaresponse}$ package is freely available from github at the address: https://github.com/Biometris/aaresponse.

The installation is done using standard $\textsf{R}$ practice—the only requirement is a working and not-too-outdated version of $\textsf{R}$. The package contains an extensive vignette showing the use of the functions in the package, using the same data as the current paper.

## Case study: the SuPro data

Here, results are presented from the SuPro study (Esser et al. 2023), an investigation comparing two proteins, bovine plasma concentrate (BP) and corn protein concentrate (CP), to whey protein concentrate (WP), which is used as the reference protein. Four men and eight women, between 55 and 70 years old, consumed the three different protein shakes with 1 week between each pair of meals, and ten blood samples were taken within 3 h after each intervention.

The raw data are stored as a table with columns for $\texttt {Participant}$, $\texttt {Period}$, $\texttt {Time}$, $\texttt {Intervention}$, columns for different individual amino acids and, possibly, aggregated aminoacid totals. For illustration purposes, the analysis here focuses on the essential amino acids only.

**Fig. 3:**
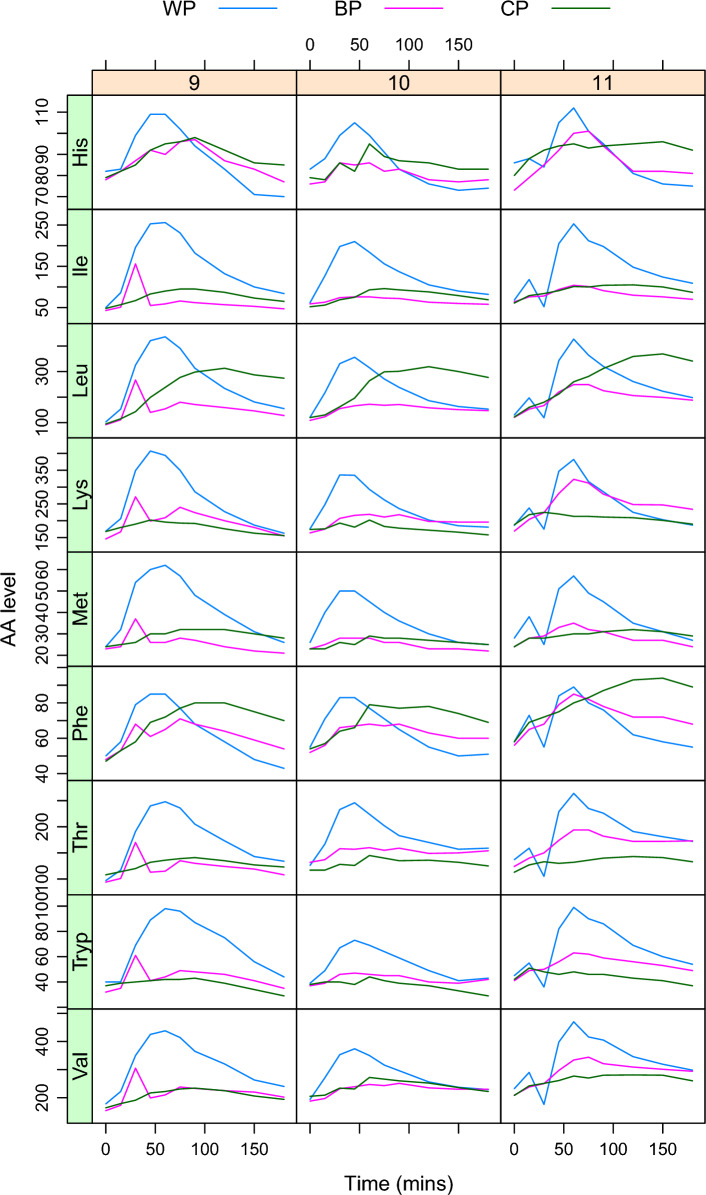
Visualizing the raw data in $\textbf{aaresponse}$. The nine essential amino acids (rows in the figure) are shown for three participants: 9, 10, and 11. Abbreviations: see text

One example of the raw data visualizations provided by the $\textbf{aaresponse}$ package is shown in Fig. 3, concentrating the essential amino acids in three participants. It is clear that the three proteins give rise to different responses, and also that there are differences between the participants. Curves for the non-essential amino acids show very similar patterns, indicating that at this timescale the variation in blood levels is dominated by the protein meal, something that is consistently the case in all data sets we have examined so far.

**Fig. 4:**
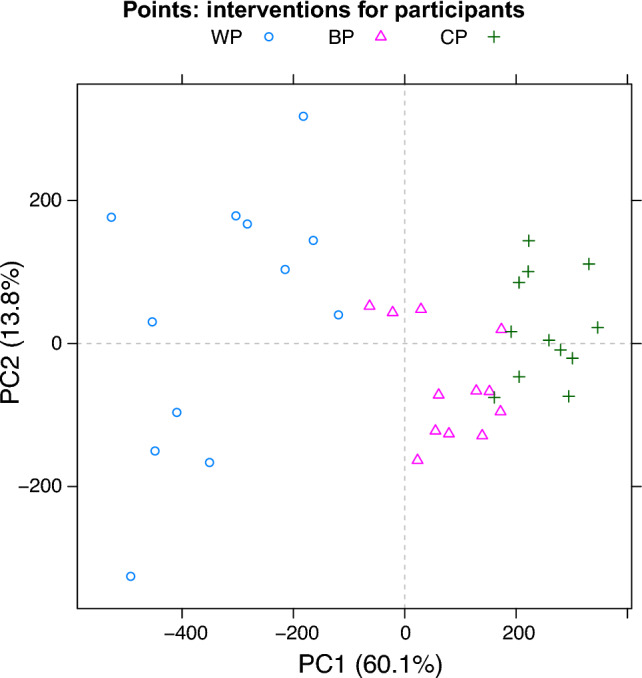
PCA score plot: the 36 symbols correspond to the 12 participants having had one of the three meals. The differences between the three interventions dominate the first PC

As an additional visualization tool, the $\textbf{aaresponse}$ package offers several different ways of applying PCA to the data. The differences lie in the way the data are organized. One extreme is to create a matrix where each row contains all time courses for a participant, and the other extreme is to simply put all time courses (one AA from one participant having had one protein meal) in separate rows. An example of an intermediate approach is to define each row as the concatenation of the time courses for a particular participant–protein combination. The PCA score plot for the latter approach is shown in Fig. 4: each symbol represents the time course for a participant having had a particular protein meal. Clearly, one can see that the three proteins show consistent differences in uptake—the effect is the dominant factor and completely determines the first PC. At the same time, the variation between participants, in this case comprising the effects on all amino acids simultaneously is still appreciable. In this case, each time course was centered individually, so that differences in baseline are eliminated to a large extent; other options are available, too.

**Fig. 5:**
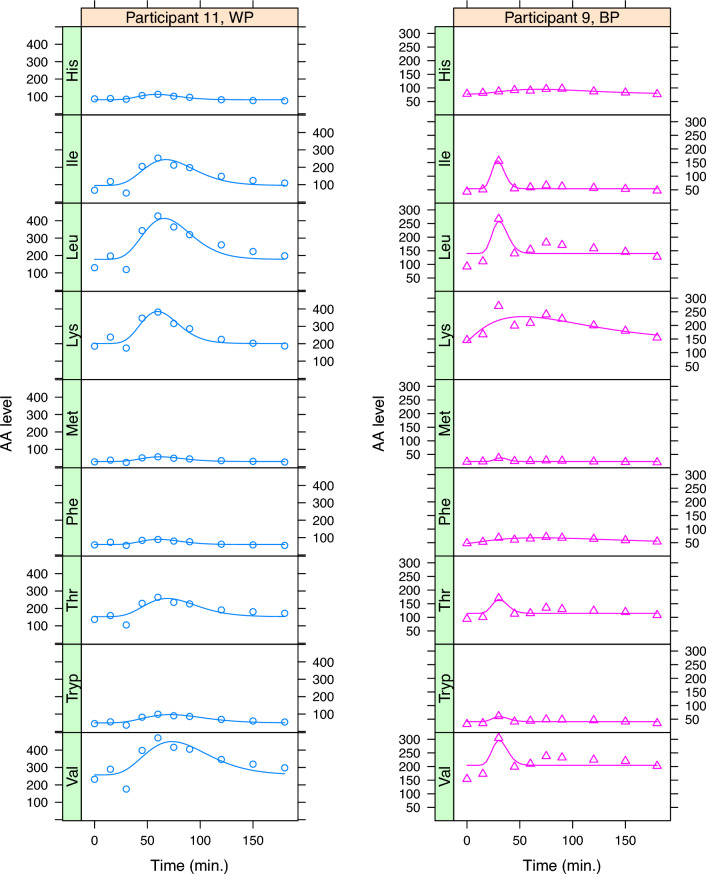
Data and fitted curves for participants 9 and 11 in Figure 3, $\texttt {WP}$ and $\texttt {BP}$, respectively

Figure 5 highlights the benefits as well as the limitations of using curve fits to estimate PoIs. The figure shows fitted curves for the WP and BP data of two of the participants from Fig. 3. In the left panels, one can immediately recognize that the curves around the third time point are less close to the data points. The effect of these deviations on the PoIs, however, is mostly limited: $\texttt {Height}$ and $\texttt {Time2Max}$ are almost unchanged. For $\texttt {AUC}$ the effects are larger, since the outlying point leads to peaks that seem to start later, and therefore have a lower surface area. With other approaches, this could even be much more extreme: when using the trapezoid rule for determining AUC values, for example, it is not uncommon to stop the integration as soon as a value drops below the first data point which in most of the examples in Fig. 5 would lead to extremely small values. Curve fit-based PoIs are more robust, but even so it could be appropriate to consider removing such points from the data, especially when the same effect is visible in several other amino acids as well, suggesting a problem at the sample level.

The right panels show a case in point: also there we can see outlying values at the third time point in several AAs, but because this is in the positive direction the curve fit algorithm tries to fit a very narrow curve through the outlying point alone in several cases. Here, one could definitely consider to remove the whole time point—if one decides to keep it in, the subsequent curation stages will most likely remove all narrow fits. This curation prevents such outlying points from causing damage, but it also decreases the number of data points in the final comparison, and hence the power of the analysis. In that sense, eliminating a single time point would be preferable. Obviously, such manual curation steps should be used sparingly, with caution, and be well documented. Since in this paper the onus is on presenting the methodology, here we will leave both time points unchanged, and consider the complete data set in the analysis.

**Fig. 6:**
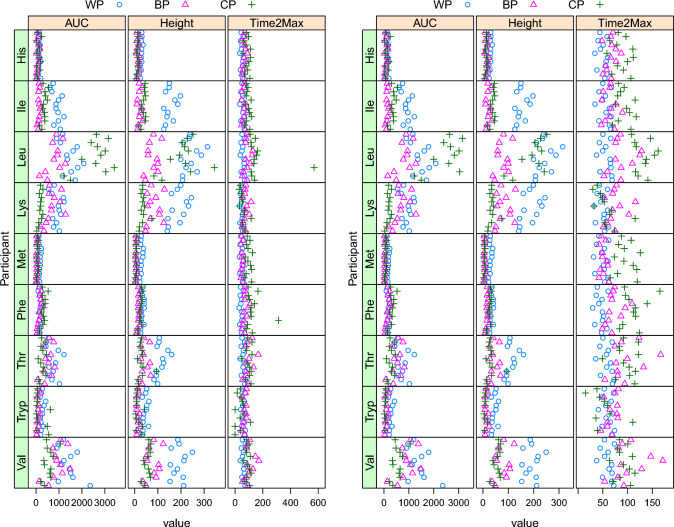
Uncurated (left) and curated (right) estimates for PoIs (AUC, Height, Time2Max) for the essential amino acids also shown in Fig. 3. In each panel, results for all participants are shown

Fitted peak parameters can, as already mentioned, be curated. Since the curve parameters do not have obvious regions in which one could expect “good” results to fall, it is wise to assess this on a case-by-case base and use visualization. Typically, the boundary values can be set by plotting the parameter distributions over all curves—all values outside the “normal” areas could be replaced with $\texttt {NA}$. This would also lead to $\texttt {NA}$ estimates for the corresponding PoIs. One would expect only a very small fraction of all values to be curated.

The curation of PoIs is easier, since these are directly interpretable, and the effects of the curation are shown in Fig. 6. The left panel shows estimated PoIs for the essential amino acids from Fig. 3—the right panel shows the same values, after curation using package defaults (positive AUC values, peak heights between 0 and 1000, and peak times between 15 and 200 min). Note that now PoIs are shown for all participants (*y* axis) and all three proteins. In both $\texttt {Leu}$ and $\texttt {Phe},$ it is easy to see a removed point in the $\texttt {Time2Max}$ column, leading to a much narrower range for the curated data. Note that similar plots are available to assess the effect of the curation of the curve fit parameters.

The (curated) results for the PoI estimates in Fig. 6 already show clear patterns: CP usually shows a later peak than BP, and WP is the protein that shows the fastest uptake; in addition, WP usually shows the highest uptake (AUC as well as Height), although $\texttt {Leu}$ seems to be an exception. The uptake for $\texttt {Phe}$ is low in absolute terms—note that this is also influenced by the protein compositions of the three protein concentrates.

**Fig. 7:**
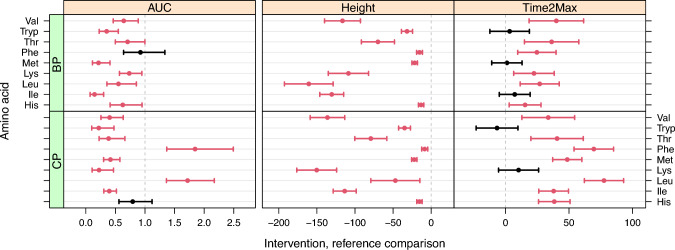
Estimates and 95% confidence intervals for the comparison of BP and CP proteins with reference WP, all PoIs, and essential amino acids only. The vertical dashed lines correspond to the “no difference” situation (1 for the AUC ratio, 0 for the differences in Height or Time2Max). Significant differences (at the 95% level) are indicated in red, others in black

Finally, the confidence intervals for all PoI comparisons (with WP as a reference) can be presented in a simple graphic such as the one shown in Fig. 7. Significant effects, not including the null hypothesis value indicated with a vertical dashed line in the confidence interval, are indicated in red; otherwise black is used. BP leads to lower AUC values for the essential amino acids, although the effect is not significant for Phe. CP shows two amino acids with increased AUCs, and a significantly lower AUC for most others. Clearly, all peak heights are significantly lower in the test proteins than in the reference protein. Nearly all estimates for the time to the peak maximum are larger in the test proteins than in the reference, although not all differences are significant. Plots like these give an immediate overview of all results, and although differences between amino acids will exist, sometimes leading to a confusing picture, general patterns should be quite obvious. It is worth noting that not all CIs are equally wide. This is the result of several effects. For one, the variation in the data is not constant—here, this is the dominating effect. In addition, if no imputation has been performed (as in the current example), the number of values taken into account may not be equal. In the current data set, very few missing values are present, and imputation would lead to virtually the same CIs as the ones shown here.

## Discussion and conclusions

Performing crossover experiments in which postprandial amino acid levels are determined in the blood of the participants is a relatively simple and very straightforward approach to assess digestibility characteristics of different proteins. We have described a principled strategy for analysing data from such experiments based on two main building blocks: curve fitting and linear mixed models.

The advantages of using fitted curves rather than the raw data are numerous. To start with, the influence of outlying points is greatly diminished because of the inherent smoothing characteristics associated with fitting parametric curves of a specific shape. Of course, this is no guarantee that all these problems will be avoided in the future: Fig. 5 is a case in point. Rather, the fitted curve parameters provide an additional checkpoint where the user can see whether these values are within plausible ranges, or not.

Furthermore, cases where no realistic curve can be fitted (often because there is no meaningful signal in the data; less often because of outliers or noise in the data) are easily identified, either in an automatic fashion of on the basis of visual inspection of the fits. Curves can also be fitted when the time points are not equidistant, or when the peak values have not quite returned to baseline level at the end of measurements—both cases that are problematic in approaches that are not based on parametric models. Parameters of interest, such as the location or height of the peak, can be calculated analytically.

Mixed models are used to compare the PoIs of the proteins in the experiment. In particular, methods have been implemented for comparing several test proteins with a reference protein. These comparisons have been set up in such a way as to provide maximal sensitivity, and in that sense are more flexible as well as more specific than alternative approaches like repeated-measures ANOVA: balanced data are no longer required, and specific contrasts such as the comparison to a reference intervention can be highlighted. The results of the comparisons can be presented as *p* values (suitably corrected for multiple testing) and as plots of confidence intervals. The latter are not only more easy to grasp but also provide more information: one particular advantage is that a confidence interval includes the information about the number of data points on which the comparison is based—this may be different for different amino acids and amino acid totals, depending on the number of successful curve fits and the amount of curation necessary. Although the functions in $\textbf{aaresponse}$ are primarily focused on comparing different protein meals, many other statistical analyses can be performed.

All of this has been implemented in an open-source $\textsf{R}$ package, $\textbf{aaresponse}$, which will continue to be developed as we apply it in more and more scientific projects. The package also provides several visualization methods—one thing to avoid is to see this as a push-button solution that automatically provides the required answers. In practice, it is a tool for the scientist that provides support for the many decisions to be taken on the route to the final outcome, concerning, e.g. removal of outlying points, detection of strange patterns, and quality control checks throughout the analysis process. In no way do we mean to imply that this is the end-all solution for the analysis of crossover experiment data investigating protein uptake, but we do hope that the $\textsf{R}$ package will prove useful for the scientific community, and will form a focal point to which other researchers can contribute their views and suggestions.

Finally, it should be noted that this methodology is not just applicable to levels of amino acids in blood after a protein meal. Similar questions are ubiquitous in food science and can also relate to, e.g., glucose or fat levels. As long as the time courses that are measured after an intervention cover enough of the response curve to obtain meaningful fits, the methodology in principle can be applied. One requirement needs to be checked: if the changes in the levels of the compound of interest change significantly during the time course due to other causes than the intervention alone, the analysis will likely not lead to useful results. That, however, is true whatever method is used for the analysis of the data. We have tried to implement the package as generally as possible, and welcome any feedback from package users.

## Data Availability

The aaresponse package contains all data necessary to reproduce the results in this paper.

## Footnotes

1 This is often referred to as the incremental AUC, or iAUC.
